# Comparison of GnRH Agonist, GnRH Antagonist, and GnRH Antagonist Mild Protocol of Controlled Ovarian Hyperstimulation in Good Prognosis Patients

**DOI:** 10.1155/2015/385049

**Published:** 2015-03-17

**Authors:** Martin Stimpfel, Eda Vrtacnik-Bokal, Barbara Pozlep, Irma Virant-Klun

**Affiliations:** Department of Obstetrics and Gynaecology, University Medical Centre Ljubljana, Slajmerjeva 3, SI-1000 Ljubljana, Slovenia

## Abstract

The reports on how to stimulate the ovaries for oocyte retrieval in good prognosis patients are contradictory and often favor one type of controlled ovarian hyperstimulation (COH). For this reason, we retrospectively analyzed data from IVF/ICSI cycles carried out at our IVF Unit in good prognosis patients (aged <38 years, first and second attempts of IVF/ICSI, more than 3 oocytes retrieved) to elucidate which type of COH is optimal at our condition. The included patients were undergoing COH using GnRH agonist, GnRH antagonist or GnRH antagonist mild protocol in combination with gonadotrophins. We found significant differences in the average number of retrieved oocytes, immature oocytes, fertilized oocytes, embryos, transferred embryos, embryos frozen per cycle, and cycles with embryo freezing between studied COH protocols. Although there were no differences in live birth rate (LBR), miscarriages, and ectopic pregnancies between compared protocols, pregnancy rate was significantly higher in GnRH antagonist mild protocol in comparison with both GnRH antagonist and GnRH agonist protocols and cumulative LBR per cycle was significantly higher in GnRH antagonist mild protocol in comparison to GnRH agonist protocol. Our data show that GnRH antagonist mild protocol of COH could be the best method of choice in good prognosis patients.

## 1. Introduction

There are several ways how to perform the controlled ovarian hyperstimulation (COH) in patients included in the in vitro fertilization program and each one has its advantages and disadvantages. Development of suitable GnRH agonists in the 1980s represented the major progress in the field [1, 2]. The most important characteristic of GnRH agonists is prevention of premature LH surge in COH through desensitization of pituitary, which helps to increase the number of retrieved oocytes and decrease the number of cancelled cycles [1]. On one side, this is a good property, but, on the other side, it can lead to the ovarian hyperstimulation syndrome (OHSS) or some other complications and side effects [3]. Due to these deficiencies of GnRH agonists, development of GnRH antagonists represented a major breakthrough because they cause less side effects [4, 5]. GnRH antagonists also reduce FSH/LH secretion and in this way they prevent LH surges although their mode of action is opposite to that of GnRH agonists. GnRH agonists bind to their receptor on pituitary and with maintaining the signal they cause desensitization of pituitary and consequently the downregulation of gonadotropin secretion after prolonged time [6]. Also GnRH antagonists bind to the receptor on a pituitary but they block it almost straight away and consequently cause the suppression of gonadotropin secretion within a few hours [7]. There are several variations in the protocol of COH using each of the GnRH analogue, but, to simplify, in the conventional long protocol the GnRH agonists are applied from 7 days before menstruation, while GnRH antagonists are applied on a fixed day of ovarian stimulation or when the size of the leading follicle is 14 mm [8]. In the last years also so called mild protocol of COH was introduced into clinical practice, in which the exogenous gonadotropins are administered at lower doses for a shorter duration in a combination with GnRH antagonists, antiestrogens, or aromatase inhibitors by definition of the International Society for Mild Approaches in Assisted Reproduction (ISMAAR) [9]. The advantages of such approach are especially in lower dose of used gonadotropins (consequently more kind to patients and lower costs) and less side effects without impairment of cumulative pregnancy rate. In spite of that, the number of retrieved oocytes and proportion of cycles with embryo cryopreservation seem to be lower [10].

Although the question about the mechanism of GnRH agonists and GnRH antagonists action is well answered, there is still no clear answer about which analogue gives better results in clinical practice. The reports are contradictory [11–18] and often favor one type of the analogue. In addition, there is still no generally accepted consensus on how to stimulate the ovaries of good prognosis patients at the beginning of their in vitro fertilization treatment. For this reason, we retrospectively analyzed the data from IVF (classical IVF and ICSI cycles together) carried out at our centre during years 2010–2013 in good prognosis patients to elucidate which protocol of COH is optimal for these patients. Because most of the reports usually include only comparison of two studied COH protocols, we included in our analysis the data obtained from three different protocols: mild protocol (cotreatment with GnRH antagonist), conventional GnRH agonist, and conventional GnRH antagonist protocol of ovarian stimulation. We comparatively analyzed the main outcomes of COH protocols, such as number of retrieved and fertilized oocytes, embryos, cryopreserved embryos, the proportion of cycles with embryo freezing and the number of cryopreserved embryos, and the clinical outcome in terms of pregnancy rate, live birth rate (LBR), and cumulative LBR.

## 2. Methods

### 2.1. Patients

In this retrospective study the data from 2373 in vitro fertilization cycles conducted at IVF Unit University Medical Centre Ljubljana from January 2010 to December 2013 in good prognosis patients were analyzed. The GnRH antagonist mild protocol of ovarian stimulation was carried out in 166 cycles, GnRH antagonist protocol in 1096 cycles, and GnRH agonist protocol in 1111 cycles. For cumulative live birth rate the births which occurred up to and including September 2014 were taken into account. Good prognosis patients were defined as patients aged ≤38 years who were undergoing the first or the second cycle of in vitro fertilization and with more than 3 oocytes retrieved regardless of their indication of infertility.

### 2.2. GnRH Antagonist Protocol of COH

In the GnRH antagonist group, a starting daily dose of 200–225 IU recombinant FSH (follitropin alfa, Gonal F Merck Serono, or follitropin beta, Puregon MSD) was started on day 2 of the menstrual cycle. Women received the GnRH antagonist cetrorelix acetate (Cetrotide; Asta Medica AG, Frankfurt, Germany) at a dose of 0.25 mg per day from the day when the dominant follicle reached a mean diameter ≤ 14 mm until the day of HCG administration. In the stimulation period rFSH daily dose was adjusted individually.

### 2.3. GnRH Antagonist Mild Protocol of COH

In the GnRH antagonist mild group, a starting daily dose of 200–225 IU (follitropin alfa, Gonal F Merck Serono, or follitropin beta, Puregon MSD) was started on day 5 of the menstrual cycle. Women received the GnRH antagonist cetrorelix acetate (Cetrotide; Asta Medica AG, Frankfurt, Germany) at a dose of 0.25 mg per day from the day when the dominant follicle reached a mean diameter ≤ 14 mm until the day of HCG administration.

### 2.4. GnRH Agonist Protocol of COH

In the GnRH agonist group ovarian stimulation was performed using GnRH analogues (Suprefact; Hoechst AG, Frankfurt/Main, Germany) administered from day 22 of the cycle in a daily dose of 0.6 mL (600 pg) s.c. After 14 days, pituitary desensitization was checked by *E*_2_ determination and B-mode ultrasound scan. Once the criteria for desensitization were fulfilled (*E*_2_ ≤ 0.05 nmol/L, follicles ≤ 5 mm in diameter, and endometrial thickness ≤ 5 mm), ovarian stimulation with a daily dose of 200–225 IU rFSH was started (follitropin alfa, Gonal F Merck Serono, or follitropin beta, Puregon MSD). GnRHa administration was continued until HCG administration. Later in the stimulation period daily dose of rFSH was adjusted individually.

In all groups, HCG (Pregnyl; N.V. Organon, Oss, The Netherlands) in a dose of 10,000 IU was administered when 3 or more follicles reached a diameter of 18 mm.

Oocyte retrieval in all groups was performed 36 h after HCG administration.

### 2.5. In Vitro Fertilization and Embryo Transfer

After retrieval of oocytes, classical IVF or ICSI was performed regarding the indication of infertility. IVF was performed in female indications of infertility, while ICSI was performed in male indications of infertility such as impaired semen quality, azoospermia, or immunological factor. Oocytes were fertilized and cultured until the next day in Fertilization Medium (Cook, Australia) or Universal IVF Medium (Origio, Denmark). The fertilization of oocytes (presence of two pronuclei and two polar bodies) was checked 16–18 hours after oocyte insemination or sperm microinjection. Normally fertilized (2PN) zygotes were cultured in the Cleavage Medium (Cook) or Blast Assist System Medium-1 (Origio) on days 2 and 3. On days 4 and 5, embryos were further cultured in Blastocyst Medium (Cook) or Blast Assist System Medium-2 (Origio). In patients with only one embryo or two embryos, one cleavage-stage embryo or two cleavage-stage embryos were transferred on day 3, while in patients with more embryos one blastocyst or two blastocysts (or morulae) were transferred on day 5 after prolonged culturing. In patients younger than 36 years and high quality embryos an elective single embryo transfer was performed. The surplus blastocysts were cryopreserved using BlastFreeze medium (Origio) and thawed in BlastThaw (Origio, Denmark) medium. The blastocysts that survived the freeze-thawing procedure (>50% of nondamaged cells) were transferred into the uterus in a natural cycle or after hormonal preparation.

### 2.6. Statistical Analysis

The data were analyzed according to the protocol of COH. The data from classical IVF cycles and ICSI cycles were joined for each COH protocol and the main outcomes of three different COH protocols (GnRH antagonist, GnRH antagonist mild, and GnRH agonist) were compared in terms of average number of retrieved and fertilized oocytes, average number of embryos, number of cryopreserved embryos, the proportion of cycles with embryo cryopreservation, and the clinical outcome of in vitro fertilization (pregnancy, miscarriage, live birth, cumulative live birth, and twin delivery). Pregnancy was defined as positive *β*HCG test and the cumulative live birth as the sum of births after fresh and frozen-thawed embryo replacements in the same group of cycles (until September 2014). To determine the differences between the groups Pearson's chi-squared test, *F*-test, and two-tailed *t*-test were used. The *P* values under 0.05 were recognized as statistically significant.

## 3. Results

**Figure 1:**
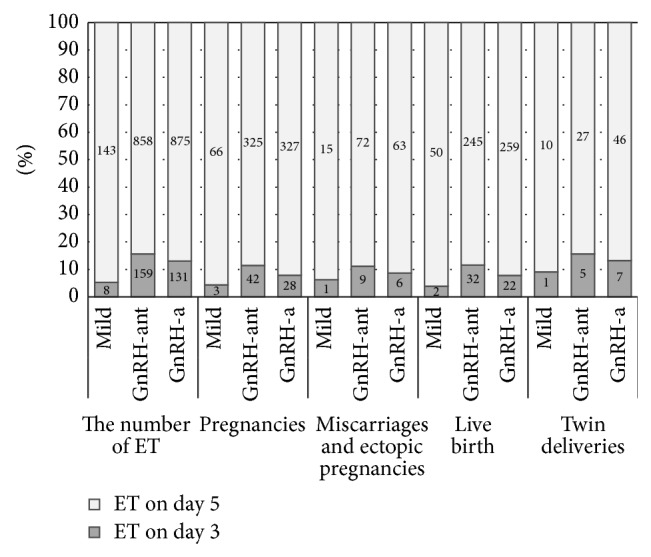
The outcome of COH in terms of pregnancies, miscarriages, births, and twin deliveries according to the day of ET (day 3 cleavage embryo or day 5 blastocyst ET) (mild: GnRH antagonist mild protocol; GnRH-ant: GnRH antagonist protocol; GnRH-a: GnRH agonist protocol).

Based on inclusion criteria, a total of 2373 cycles of in vitro fertilization were included in this study. Of all cycles, GnRH antagonist mild protocol of ovarian stimulation was carried out in 166 cycles, GnRH antagonist protocol in 1096 cycles, and GnRH agonist protocol in 1111 cycles. Altogether, as the most important outcome, 674 live births were achieved in all cycles: 610 live births after fresh embryo transfers (ET) (56 live births from day 3 ET and 554 from day 5 ET) and additional 65 live births after frozen-thawed embryo transfers (FET) (Figure 1). Overall, this means 28.4% cumulative live birth rate per cycle.

### 3.1. The Outcome of Different COH Protocols

The outcome and comparison of different protocols of COH cycles are presented in Table 1 and in Table 2. To summarize, the age of the patients and the baseline FSH were comparable in all groups. The number of retrieved oocytes (10.8 ± 5.6) was the highest and the proportion of immature oocytes was the lowest (14.1%) in GnRH agonist protocol. Also the average number of embryos was the highest in GnRH agonist protocol (5.4 ± 3.5), but the difference was significant only when compared to GnRH antagonist protocol (4.7 ± 3.3, *P* < 0.0001). On the contrary, the proportion of fertilized oocytes (57.7%) and embryos (56.3%) was the highest in GnRH antagonist mild protocol. In addition, the average number of frozen embryos per cycle (0.5 ± 1.3) and the proportion of cycles with embryo freezing (18.9%) were significantly the lowest in GnRH agonist protocol. In the most important category, in the LBR per cycle after fresh ET, the results were comparable between all groups, despite the fact that pregnancy rate per cycle (41.6%) was significantly higher in GnRH antagonist mild protocol compared to both other protocols (Table 2). At this point it is worth it to mention that cumulative LBR per cycle was significantly higher in GnRH antagonist mild protocol (35.6%) compared to GnRH agonist protocol (27.3%, *P* = 0.0275).

To determine if the day of the ET (day 3 cleavage embryo or day 5 blastocyst ET) has any influence on the outcome of COH, we additionally analyzed the data about the number of ETs, pregnancies, miscarriages, births, and twin deliveries according to the day of ET and compared them between COH protocols. The results showed (Figure 1) that there is no significant difference in LBR, twin deliveries, and miscarriages per ET between COH protocols irrespective of the day of ET. The only difference was in pregnancy rate per ET on day 5 ET, when GnRH antagonist mild (46.2%) and GnRH agonist (37.4%, *P* = 0.0455) protocols were compared. In day 3 ET group no such difference was observed.

Because the number of retrieved oocytes is significantly different between studied COH protocols (Table 1) and this could influence the cycle outcome, we divided patients based on the number of retrieved oocytes, to analyze the different outcomes according to the 3 different COH protocols. Therefore, the patients were divided to quartiles (patients with 4–6 oocytes, 7–9 oocytes, 10–13 oocytes, 14, or more retrieved oocytes) and pregnancy rates, LBR, and cumulative LBR were compared between COH protocols. Results showed that in groups of patients with 7–9 oocytes and with 10–13 retrieved oocytes there is no difference between COH protocols. The difference was observed in group of patients with 4–6 oocytes since the pregnancy rate per cycle (45.8% versus 29.5%, *P* = 0.0160), LBR (33.6% versus 23.0%, *P* = 0.0444), and cumulative LBR (37.3% versus 24.1%, *P* = 0.0388) were significantly higher in GnRH antagonist mild protocol compared to GnRH agonist protocol. Similar trend was observed also in a group of patients where 14 or more oocytes were retrieved. Despite there being no difference in pregnancy rate, the LBR was significantly higher in GnRH antagonist mild protocol (42.9%) compared to GnRH antagonist protocol (23.1%, *P* = 0.0480). Additionally, also the cumulative LBR was significantly higher in GnRH antagonist mild protocol (57.1%) compared to both GnRH antagonist (32.3%, *P* = 0.0032) and GnRH agonist protocol (33.1%, *P* = 0.0256).

## 4. Discussion

In this study we retrospectively analyzed the data from IVF program carried out at IVF Unit of University Medical Centre in good prognosis patients to elucidate which protocol of COH gives the best results at our condition. We compared GnRH antagonist, GnRH antagonist mild, and GnRH agonist protocol of COH. As already mentioned above, there is still no consensus, in which protocol of COH is optimal in good prognosis patients at the beginning of in vitro fertilization treatment. The results of Orvieto et al. [19] advocated in favor of using GnRH agonist protocol in the first IVF cycles performed in young patients, since the clinical pregnancy rate was significantly higher than in GnRH antagonist protocol. Contrarily, the meta-analysis conducted in 2011 showed that there were no significant differences between these two protocols of ovarian stimulation regarding the live birth rate [20]. Although the reanalysis of these data disclosed significantly higher live birth rate in the GnRH agonist protocol [18]. The most recent study by Grow et al. [11] was carried out in a large number of very similar population of patients than in our study and showed that the GnRH agonist protocol decreased the cancellation risk and increased embryo implantation and live birth rate in comparison with GnRH antagonist protocol of ovarian stimulation. On the contrary, in our study we did not observe any difference in live birth rate after fresh ET between all the groups of studied COH protocols, although the cumulative live birth rate was significantly higher in GnRH antagonist mild protocol when compared to GnRH agonist protocol. Other results showed that the average numbers of retrieved oocytes, embryos, and transferred embryos per ET were significantly higher after GnRH agonist than GnRH antagonist protocol, although the proportion of cycles with embryo cryopreservation was significantly higher in GnRH antagonist protocol. The recent meta-analysis by Xiao et al. showed similar results, since there were significant differences in the number of retrieved oocytes and pregnancy rate between GnRH agonist and GnRH antagonist protocol, although there were no differences in live birth rate. The reason why some propose that GnRH agonists are more appropriate in good prognosis patients could lay in the endometrial receptivity. Studies by Orvieto et al. [21] and Huang et al. [17] showed that in GnRH agonist protocol the endometrium is thicker, although some other studies show that there is no difference [12, 13]. Contradictory are also results of studies, where researchers tried to determine biomarkers of endometrial receptivity. Simon et al. [22] showed that endometrial gene expression is more similar to the natural cycle, when GnRH antagonist was applied for COH, while in a study by Ruan et al. [23] the results oppositely showed that two biomarkers related to the endometrial receptivity, integrin beta-3 and leukaemia-inhibitory factor, were correlated with the higher implantation rate after COH using GnRH agonist. On the other hand, all these explanations are also not in accordance with the results of our study, since the cumulative live birth rate per cycle was significantly higher in GnRH antagonist mild protocol when compared to GnRH agonist protocol, although the number of retrieved oocytes was significantly lower after GnRH antagonist mild protocol of ovarian stimulation. The lower number of oocytes is often linked to poor ovarian response, but in a case of GnRH antagonist mild protocol of COH the lower number of retrieved oocytes is normal and most probably represents a homogenous group of good quality oocytes [24, 25]. This explanation could be confirmed also with the results of our study, since in the subgroup of patients with 4–6 retrieved oocytes the pregnancy rate per cycle, LBR, and cumulative LBR were significantly higher in GnRH antagonist mild protocol compared to GnRH agonist protocol. Also the study by Baart et al. [26] indirectly indicated this, since the results showed that both mild and conventional protocols of ovarian stimulation generated on average a similar number of chromosomally normal embryos in spite of significantly lower number of oocytes and embryos retrieved after mild protocol of ovarian stimulation. The meta-analysis showed that optimal embryo implantation was achieved when 5 oocytes were retrieved after mild ovarian stimulation and when 10 oocytes were retrieved after GnRH agonist stimulation [24]. In addition, the ongoing pregnancy rate per embryo transfer, as a function of the number of retrieved oocytes, was significantly higher after the mild protocol of ovarian stimulation [24]. Similar to our study, also some other studies showed that the outcome of the IVF/ICSI cycle is not compromised, if the mild protocol of ovarian stimulation is used in comparison with GnRH agonist protocol [25, 27, 28].

We may conclude that the GnRH antagonist mild protocol of ovarian stimulation could be the method of choice to stimulate the ovaries of good prognosis patients without a risk of compromising the outcome of IVF cycle.

## Figures and Tables

**Table 1:** The outcome of COH in terms of oocytes and embryos.

	COH protocol			Statistical significance at *P* < 0.05
	Mild protocol	GnRH antagonist protocol (GnRH-ant)	GnRH agonist protocol (GnRH-a)	
Oocyte aspirations	166	1096	1111	

Average female age (years)	31.8 ± 3.2	31.8 ± 3.4	32.0 ± 3.4	/

Baseline FSH (mIU/mL)	6.4 ± 1.8	6.7 ± 2.1	6.9 ± 2.0	/

Oocytes (per cycle)	1500 (9.0 ± 5.2)	10249 (9.4 ± 5.0)	12004 (10.8 ± 5.6)	*P* = 0.0001 (mild versus GnRH-a), *P* < 0.0001 (GnRH-ant versus GnRH-a)

Immature oocytes (%)	240 (16.0%)	1602 (15.6%)	1687 (14.1%)	*P* = 0.0420 (mild versus GnRH-a), *P* = 0.0010 (GnRH-ant versus GnRH-a)

Fertilized oocytes, 2PN (%)	865 (57.7%)	5440 (53.1%)	6235 (51.9%)	*P* = 0.0009 (mild versus GnRH-ant), *P* < 0.0001 (mild versus GnRH-a)

Embryos (%)	845 (56.3%)	5198 (50.7%)	5968 (49.7%)	*P* < 0.0001 (mild versus GnRH-ant), *P* < 0.0001 (mild versus GnRH-a)

Embryos (per cycle)	5.1 ± 3.7	4.7 ± 3.3	5.4 ± 3.5	*P* < 0.0001 (GnRH-ant versus GnRH-a)

Embryo transfers (%)	151 (91.0%)	1017 (92.8%)	1006 (90.5%)	/

Transferred embryos (per ET)	1.6 ± 0.5	1.6 ± 0.5	1.7 ± 0.4	*P* = 0.0022 (mild versus GnRH-a), *P* < 0.0001 (GnRH-ant versus GnRH-a)

Cycles with embryo freezing (%)	50 (30.1%)	317 (28.9%)	210 (18.9%)	*P* = 0.0008 (mild versus GnRH-a), *P* < 0.0001 (GnRH-ant versus GnRH-a)

Frozen embryos (per cycle)	0.8 ± 1.4	0.9 ± 1.8	0.5 ± 1.3	*P* = 0.0243 (mild versus GnRH-a), *P* < 0.0001 (GnRH-ant versus GnRH-a)

OHSS	0	4	12	/

**Table 2:** The outcome of COH in terms of pregnancies, miscarriages, and deliveries.

	COH protocol			Statistical significance at *P* < 0.05
	Mild protocol	GnRH antagonist protocol (GnRH-ant)	GnRH agonist protocol (GnRH-a)	
Pregnancies	69	367	355	

Pregnancies (per cycle)	41.6%	33.5%	32.0%	*P* = 0.0414 (mild versus GnRH-ant), *P* = 0.0141 (mild versus GnRH-a)

Pregnancies (per ET)	45.7%	36.1%	35.3%	*P* = 0.0227 (mild versus GnRH-ant), *P* = 0.0134 (mild versus GnRH-a)

Miscarriages and ectopic pregnancies (per pregnancy)	16 (23.2%)	81 (22.1%)	69 (19.4%)	/

Biochemical pregnancies	2	12	6	/

Live birth rate (per cycle)	52 (31.3%)	277 (25.3%)	281 (25.3%)	/

Twin deliveries	21.2% (11/52)	11.6% (32/277)	18.5% (53/281)	*P* = 0.0163 (GnRH-ant versus GnRH-a)

Live births after FET	7	35	23	/

Cumulative live birth rate (per cycle)	59 (35.6%)	312 (28.5%)	303 (27.3%)	*P* = 0.0275 (mild versus GnRH-a)

Cumulative twin deliveries	20.3% (12/59)	13.1% (41/312)	18.2% (55/303)	/
